# Piperine suppresses the Wnt/β-catenin pathway and has anti-cancer effects on colorectal cancer cells

**DOI:** 10.1038/s41598-020-68574-2

**Published:** 2020-07-15

**Authors:** Gracielle C. de Almeida, Luiz F. S. Oliveira, Danilo Predes, Harold H. Fokoue, Ricardo M. Kuster, Felipe L. Oliveira, Fabio A. Mendes, Jose G. Abreu

**Affiliations:** 1 Instituto de Ciências Biomédicas, Programa de Biologia Celular e do Desenvolvimento, Universidade Federal do Rio de Janeiro, Avenida Carlos Chagas Filho 373, Centro de Ciências da Saúde, Bl F sala F2-015, Cidade Universitária, Ilha Do Fundão, Rio de Janeiro, RJ CEP 21941-902 Brazil; 2 Instituto Nacional de Ciência e Tecnologia de Fármacos e Medicamentos (INCT-INOFAR; http://www.inct-inofar.ccs.ufrj.br/), Laboratório de Avaliação e Síntese de Substâncias Bioativas (LASSBio®, http://www.lassbio.icb.ufrj.br), Universidade Federal do Rio de Janeiro, CCS, Cidade Universitária, Rio de Janeiro, RJ Brazil; 3 Programa de Pós-Graduação em Farmacologia e Química Medicinal, Instituto de Ciências Biomédicas, Universidade Federal do Rio de Janeiro, Avenida Carlos Chagas Filho, 373, Ilha do Fundão, Rio de Janeiro, RJ 21941-912 Brazil; 4 Departamento de Química, Universidade Federal do Espírito Santo, Vitória, ES Brazil

**Keywords:** Colon cancer, Morphogen signalling, Target identification

## Abstract

More than 94% of colorectal cancer cases have mutations in one or more Wnt/β-catenin signaling pathway components. Inactivating mutations in *APC* or activating mutations in β-catenin (*CTNNB1*) lead to signaling overactivation and subsequent intestinal hyperplasia. Numerous classes of medicines derived from synthetic or natural small molecules, including alkaloids, have benefited the treatment of different diseases, including cancer, Piperine is a true alkaloid, derived from lysine, responsible for the spicy taste of black pepper (*Piper nigrum*) and long pepper (*Piper longum*). Studies have shown that piperine has a wide range of pharmacological properties; however, piperine molecular mechanisms of action are still not fully understood. By using Wnt/β-catenin pathway epistasis experiment we show that piperine inhibits the canonical Wnt pathway induced by overexpression of β-catenin, β-catenin S33A or dnTCF4 VP16, while also suppressing β-catenin nuclear localization in HCT116 cell line. Additionally, piperine impairs cell proliferation and migration in HCT116, SW480 and DLD-1 colorectal tumor cell lines, while not affecting the non-tumoral cell line IEC-6. In summary, piperine inhibits the canonical Wnt signaling pathway and displays anti-cancer effects on colorectal cancer cell lines.

## Introduction

The canonical Wnt signaling pathway is an important mechanism to control different phenomena since embryonic development throughout adulthood such as axis patterning, determination of tissues, stem cell renewal, cellular polarization and migration. Deregulation of this pathway causes several disorders such as cancer, neurodegenerative and neuropsychiatric diseases.

When the Wnt ligand is not bound to the receptors Frizzled and Low-density lipoprotein receptors 5/6 (LRP5/6), cytosolic β-catenin is degraded by a multiprotein complex, named destruction complex, formed by Axin, Adenomatosis polyposis coli (APC), Protein phosphatase 2A (PP2A), Glycogen synthase kinase 3 (GSK3) and Casein kinase 1α (CK1α)^1^. The phosphorylation of β-catenin by CK1α and GSK3 marks it for ubiquitination and posterior degradation by the proteasomal machinery^2^. The binding of Wnt ligands to its receptors triggers the signaling cascade that disassembles the β-catenin destruction complex^3^. Wnt signaling activation induces Disheveled (Dvl) mediated Axin membrane translocation, which binds to the cytoplasmic portion of LRP5/6^3–5^. Due to the membrane position of the degradation complex, GSK3 and CK1α start to phosphorylate LRP5/6 leading to β-catenin stabilization in the cytoplasm^6^. In that sense, β-catenin stabilization also occur through Axin proteolysis catalyzed by Tankyrases (TNKs)^7^. Then, β-catenin translocates to the nucleus where it will bind TCF/LEF transcription factors.

One of the best-characterized mechanisms controlled by Wnt signaling is the cellular proliferation in the intestinal crypt and maintenance of intestinal stem cells. Mutations in APC or β-catenin (CTNNB1) genes result in Wnt signaling overactivation and promote intestinal hyperplasia^8^. Alternatively the presence of the Wnt extracellular inhibitor, DKK1, or the deletion of the TCF4 results in Wnt signaling blockage, leading to a failure in the normal development of proliferative crypts^9,10^. The finding that APC could negatively regulate β-catenin provided further support for the connection between Wnt and cancer^11,12^. According to the Cancer Genome Atlas more than 94% of cases of colorectal cancer have mutations in at least one component of the Wnt signaling pathway^13^. Hence, inhibiting the pathway downstream of these gain of function mutations is an interesting strategy to impair tumor progression.

The treatment of different diseases, including cancer, has benefited from numerous classes of drugs isolated from plants natural products. Many interesting small molecules with biological activity to treat diseases have been published in recent years^14–16^. Our group uncovered the mechanism of action of distinct flavonoids on targeting Wnt signaling^17–19^.

Among the natural substances of alkaloid origin with pharmacological properties, piperine is outstanding. This substance is an example of a true alkaloid, derived from lysine, responsible for the spicy taste of black pepper (*Piper nigrum*) and long pepper (*Piper longum*), originally from the family *Piperaceae*^20,21^. Studies have shown that piperine has diverse pharmacological properties, including anticonvulsive activity^22^, antioxidant activity^23^, anti-inflammatory^24,25^, liver protective^26^, neuroprotective^27^ and acts as an antimicrobial agent^28^. Piperine also exhibits potential to treat depressive disorders and to enhance memory in animal models^29,30^. In addition to all these properties, piperine also exerts an anticancer effect^31^.

Its effects have been reported for different cancer types. In lung cancer, piperine induces apoptosis via p53 signaling^32^. In breast cancer, this substance suppresses proliferation and metastasis both in vitro and in vivo^33^. In melanoma cells, piperine inhibitis NF-κβ, c-Fos, ATF-2 and CREB^34^. Piperine inhibits proliferation, causes cell cycle arrest and induces autophagy in prostate cancer cell lines^35^. In rectal cancer, piperine increases reactive oxygen species leading to apoptosis increase^36^. Additionally, some effects of piperine on colorectal cancer were reported, such as inhibition of cell proliferation and activation of apoptotic program by endoplasmic reticulum stress^37^. However, piperine molecular mechanisms of action on colorectal cancer have not been elucidated, as well as its relation to the Wnt signaling pathway.

Thus, this work aims to molecularly characterize the alkaloid piperine inhibitory effects on the Wnt signaling pathway, and its effects on colorectal cancer biological properties.

## Results

### Piperine inhibits the canonical Wnt pathway at the level of TCF

**Figure 1:**
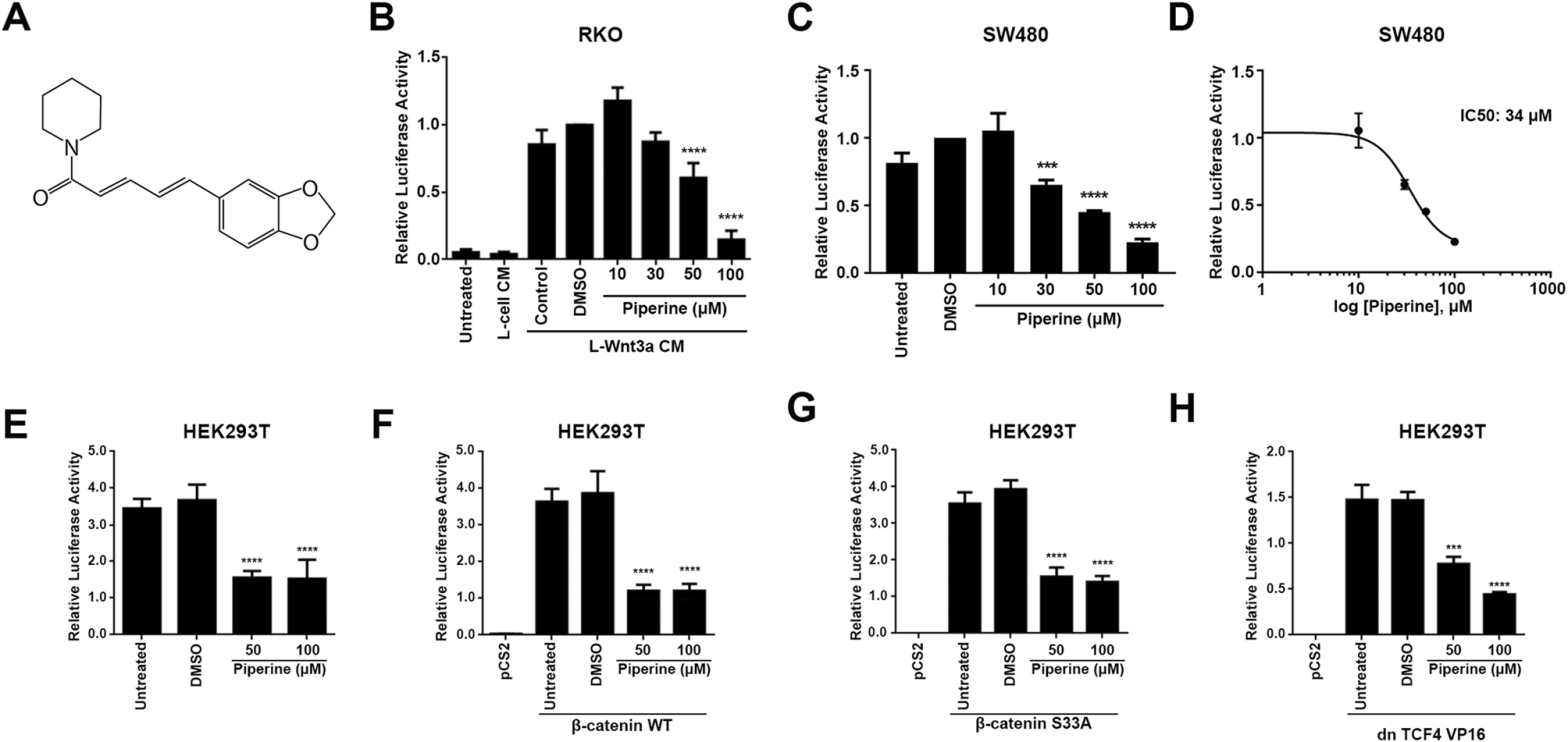
Piperine inhibits TCF/LEF induced transcription. (**A**) Molecular structure of piperine. (**B**) Relative luciferase activity of RKO pBAR/*Renilla* cells treated or not with different concentrations of piperine and L-Wnt3a conditioned medium. (**C**) Relative luciferase activity of SW480 pBAR/*Renilla* cells treated or not with different concentrations of piperine. Piperine inhibits Wnt signaling on both cells that have normal (RKO) or overexpressed (SW480) Wnt signaling. (**D**) Relative luciferase activity of HEK293T cells transfected with (**E**) pCS2, (**F**) β-catenin WT, (G) β-catenin S33A or (H) dnTCF4 VP16 and treated or not with different concentrations of piperine. ***p < 0.001, ****p < 0.0001.

In order to determine piperine effects on Wnt/β-catenin signaling we used two different Wnt reporter colorectal tumor cell lines, RKO pBAR/*Renilla* and SW480 pBAR/*Renilla*. Since RKO pBAR/*Renilla* cells do not have mutations on Wnt signaling components, incubation with L-Wnt3a conditioned medium (Wnt3a CM) with 0.2% DMSO resulted in 12-fold increase in luciferase activity. Treatment of RKO pBAR/*Renilla* cell line with 50 or 100 μM piperine decreased the luciferase activity by 40% and 85%, respectively (Fig. 1B), while treatment with 10 or 30 μM piperine had no effect on reporter activity. Piperine treatment of SW480 pBAR/*Renilla* cell lines at 30, 50 and 100 μM inhibited the Wnt reporter luciferase activity by 30%, 50% and 75%, respectively (Fig. 1C). SW480 cell line harbors an *APC* gene deletion, thus expressing a truncated form. For this reason, Wnt/β-catenin in the SW480 cell line is constitutively active. We determined piperine half maximal inhibitory concentration (IC50) as 34 μM by nonlinear regression of previous SW480 pBAR/*Renilla* data means (Fig. 1D).

In order to elucidate at which level of the Wnt/β-catenin signaling cascade piperine acts, as well as its mechanism of action, we performed an epistasis assay by measuring Wnt signaling luciferase reporter gene. Since piperine inhibited Wnt/β-catenin signaling on SW480 cell line, its mechanism of action should be downstream of APC, and independent of the destruction complex. For solving this mechanism, HEK293T cells were co-transfected with TOP-Flash and CMV-*Renilla* reporter plasmids together with the empty vector pCS2, wild type β-catenin, β-catenin S33A (constitutively activated form) or dnTCF4 VP16 (constitutively activated form, independent of β-catenin binding). Piperine treatment at 50 and 100 μM inhibited the Wnt signaling reporter activity basal levels of pCS2 transfected HEK293T cells by 60% (Fig. 1E). Treatment with the same piperine concentrations inhibited Wnt signaling induction by 70% and 65% of wild type β-catenin and S33A β-catenin HEK293T transfected cells, respectively (Fig. 1F, G). Finally, 50 and 100 μM piperine decreased the Wnt/β-catenin signaling reporter induction of HEK23T cells transfected with the constitutive active form of TCF4, dnTCF4 VP16 by 53% and 67%, respectively (Fig. 1H). These data show that piperine inhibits Wnt signaling downstream of β-catenin stabilization, probably by impairing TCF binding to DNA, or to the transcriptional machinery.

### Piperine reduces β-catenin nuclear localization

**Figure 2:**
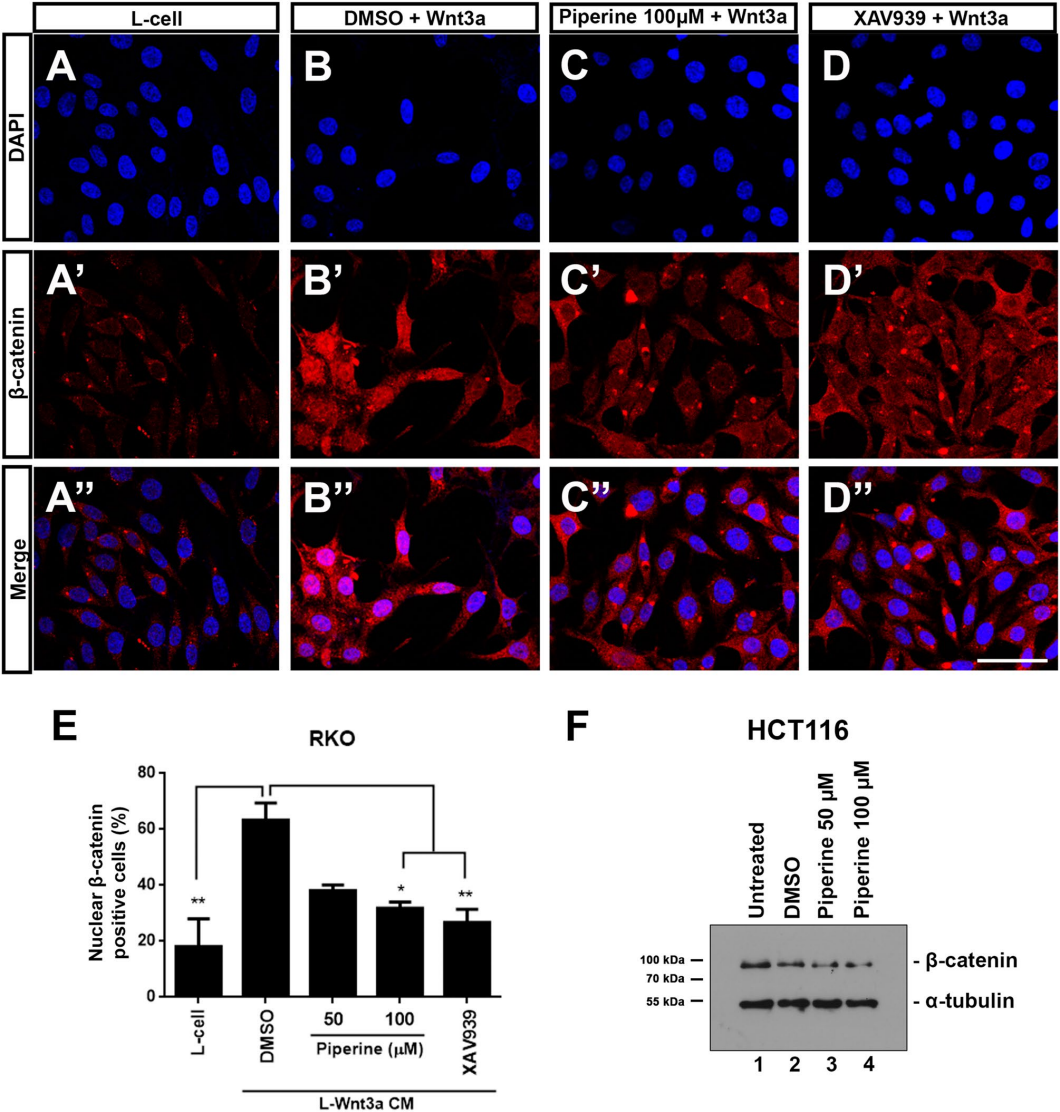
Piperine reduces β-catenin nuclear localization. Immunostainings of β-catenin of RKO cells treated with (**A**–**A**″) L-cell conditioned medium, with (**B**–**B**″) L-Wnt3a conditioned medium co-treated with DMSO or with (**C**–**C**″) piperine 100 µM. (**D**–**D**″) XAV939 was used as a positive control for Wnt signaling inhibition. (**E**) Graph of β-catenin positive nuclei percentage quantification. (**F**) Immunoblot for β-catenin of HCT116 cells untreated or treated with DMSO or 50, 100 μM piperine for 24 h. The raw immunoblot data is shown in Supplementary Figure S4. Scale bar = 38 μm. *p < 0.05, **p < 0.01.

To determine if piperine inhibits Wnt signaling by impairing β-catenin nuclear localization we incubated RKO cells with Wnt3a CM treated with 0.2% DMSO and 50 or 100 μM piperine for 24 h. After treatment, RKO cells were fixed for β-catenin immunocytochemistry staining assay. 50 and 100 μM piperine inhibited the nuclear β-catenin positive cell count compared to the DMSO control by approximately 50% (Fig. 2B-E). As a control inhibitor we used 10 μM XAV939, a commercial TNKS inhibitor that decreases β-catenin stabilization and, consequently, its nuclear translocation (Fig. 2D). For testing if piperine impairs β-catenin stabilization, we incubated HCT116 cells with 50 or 100 μM piperine for 24 h and then harvested the cell lysate for β-catenin detection through immunoblot assay. Piperine treatment had no dramatic effect on β-catenin total levels in both conditions compared to DMSO control, suggesting that piperine has no effect on β-catenin stabilization (Fig. 2F).

### Piperine diminishes colorectal cancer cells viability

**Figure 3:**
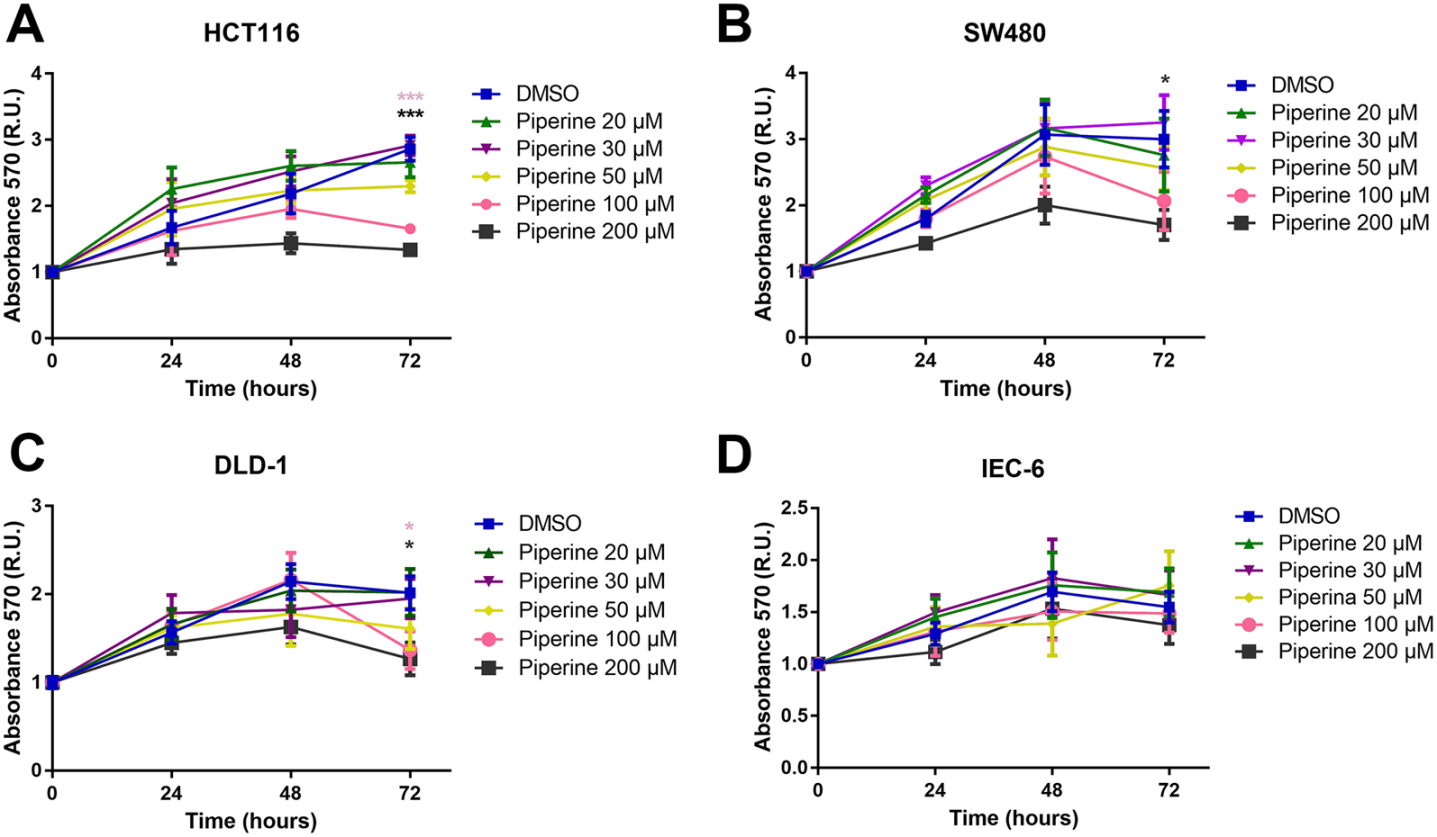
Piperine does not affect viability of non-tumoral intestine cell line IEC-6. Graphs show MTT assay absorbance levels in (**A**) HCT116, (**B**) SW480, (**C**) DLD-1 and (**D**) IEC-6 cell lines, after 24, 48 or 72 h of treatment with 20, 30, 50, 100 or 200 μM piperine. *p < 0.05, ***p < 0.001.

Since piperine impairs the canonical Wnt signaling in colorectal cancer cell lines, we next asked if piperine decreases viability of these cell lines. For this reason, we treated the colorectal cancer cell lines HCT116, SW480 and DLD-1, as well as the non-tumoral intestine cell line IEC-6 with 20, 30, 50, 100 and 200 µM piperine for 24, 48 and 72 h. After treatment, we analyzed cell viability by MTT assay. Treatment of 100 μM and 200 μM piperine decreased the viability of HCT116 and SW480 cells by 45 and 44% in 72 h (Fig. 3A,C). Treatment of 200 μM piperine decreased the viability of DLD-1 cells by 56% only after 72 h (Fig. 3B). Treatment of 200 μM piperine decreased RKO cells viability in 24, 48 and 72 h, while 100 μM piperine decreased its viability in 72 h (Supplementary Figure S1G). IEC-6 cells viability was not affected (Fig. 3D). It is notable that piperine did not decrease cell viability of colorectal cancer cell lines with constitutively active Wnt signaling in every tested concentrations before 72 h.

### Piperine impairs colorectal cancer cell lines proliferation

**Figure 4:**
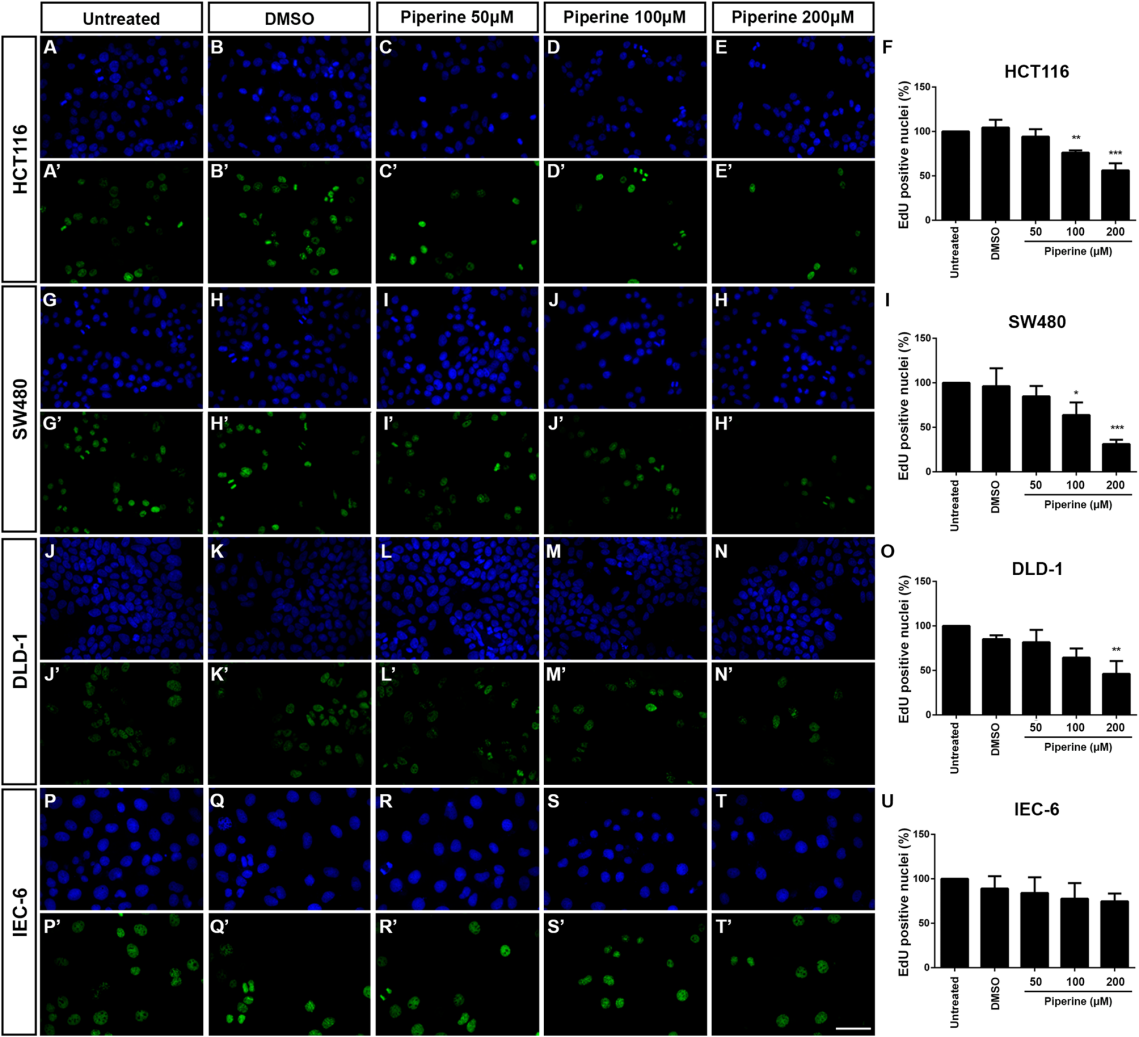
Piperine decreases colorectal cancer cell lines proliferation. Immunocytochemistry showing DAPI staining of (**A**–**E**) HCT116, (**G**–**H**) SW480, (**J**–**N**) DLD-1 and (**P**–**T**) IEC-6, and EdU staining of (**A**′–**E**′) HCT116, (**G**′–**H**′) SW480, (**J**′–**N**′) DLD-1 and (**P**′–**T**′) IEC-6. Cells were treated with DMSO, 50, 100, 200 μM piperine, or untreated according to label. Quantification of the percentage of EdU positive nuclei of (**F**) HCT116 cells, (**I**) SW480, (**O**) DLD-1, (**U**) IEC-6 cells treated or not with 50, 100 or 200 µM piperine. *p < 0.05, **p < 0.01, ***p < 0.001. Scale bar = 50 μm.

Considering that piperine impairs Wnt/β-catenin signaling in colorectal cancer cell lines without decreasing cell viability, we next asked whether piperine would halt cell proliferation. In order to test that, we performed the Click-iT EdU assay using HCT116, SW480, DLD-1 and IEC-6 cell lines treated with 50, 100 or 200 μM piperine for 24 h. Piperine decreased EdU positive cell count of every colorectal cancer cell line tested (Fig. 4). 100 and 200 μM piperine decreased the EdU positive HCT116 cell count by 27% and 46%, respectively (Fig. 4A–F,A′–E′) and decreased EdU positive DLD-1 cell count by approximately 35% and 68%, respectively (Fig. 4J–O,J′–N′). Furthermore, 200 μM piperine decreased the SW480 EdU positive cell count by approximately 55% (Fig. 4G–I,G′–H′). In contrast, piperine did not affect IEC-6 non-tumoral intestine cell line proliferation (Fig. 4P–U,P′–T′). In order to test whether piperine impairs proliferation in a cell line that does not harbor a Wnt signaling gain of function mutation, we treated RKO cells under the same conditions (Supplementary Figure S1A–F). 200 μM piperine treatment decreased by 36% the EdU positive RKO cell count. In addition, we generated a HEK293T *CTNNB1* KO cell line (Supplementary Figure S1Z′), in order analyze the piperine treatment impact on proliferation in comparison to the HEK293T WT cell line (Supplementary Figure S1M–Z). Both 200 μM piperine and 10 μM XAV939 reduced by 75% and 42% the EdU positive cell count of the WT cell line, but did not decrease the proliferation of the *CTNNB1* KO cell line. These data show that piperine suppresses colorectal cancer cell lines proliferation, without affecting the non-tumoral intestine cell line proliferation. Additionally, it suggests that piperine effect on cell proliferation relies partially on increased Wnt signaling activity.

**Figure 5:**
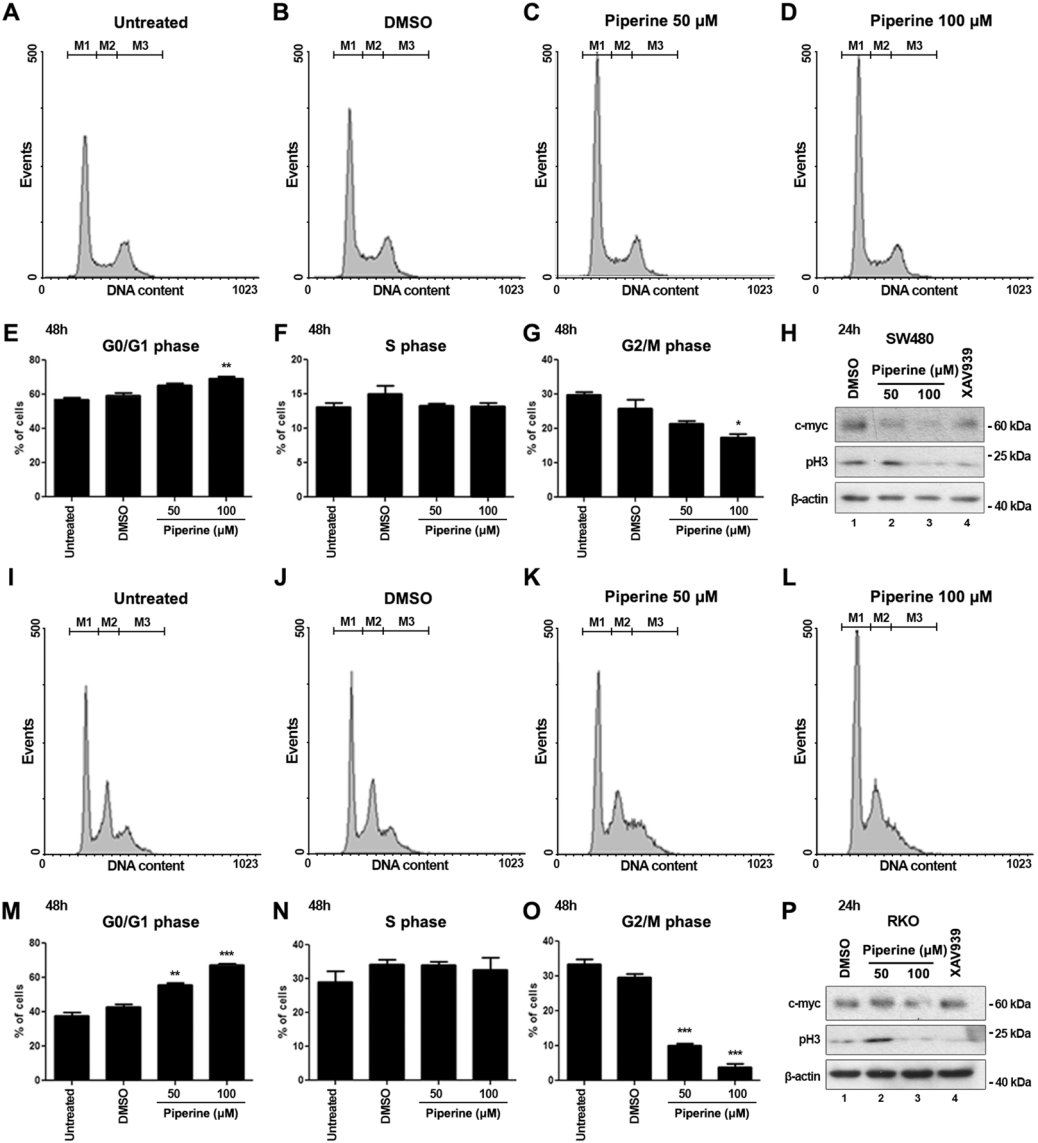
Piperine impairs cell cycle progression of SW480 and RKO cell lines. (**A**–**D**) Histograms represent DNA content of cells in G0/G1 (gate M1), S (gate M2) and G2/M phase (gate M3) of the cell cycle of SW480 cells untreated or treated with DMSO, 50 and 100 μM piperine for 48 h. (**E**) G0/G1 (**F**) S and (**G**) G2/M phase contents graphs of treated SW480 cells. (**H**) Western blot for analysis of c-myc and pH3 protein contents of SW480 cells treated with DMSO, 50 or 100 μM piperine, or 10 μM XAV939 for 24 h. β-actin was stained as a loading control. (**I**–**L**) Histograms of RKO cells untreated or treated with the described conditions. (**M**) G0/G1 (**N**) S and (**O**) G2/M phase contents graphs of treated RKO cells. (**P**) Western blot of treated RKO cells. These data are representative of three independent experiments. The raw immunoblot data is shown in Supplementary Figure S4. *p < 0.05; **p < 0.01; ***p < 0.001.

We next wonderer whether the proliferation inhibition by piperine treatment leads to cell cycle arrest. In order to investigate whether piperine interferes with cell cycle progression of RKO and SW480 cells, both cell lineages were treated with 50 or 100 μM piperine for 48 h and the cell count percentage in each cell cycle phase was monitored and quantified according to DNA content. Piperine treatment for 48 h increased the percentage of SW480 cells in G0/G1 phase (Fig. 5A-E—gate M1), and decreased the percentage of SW480 cells in G2/M phase of cell cycle (Fig. 5G—gate M3). However, piperine did not alter SW480 cell count percentage in S phase (Fig. 5F—gate M2). Western blot of SW480 cells treated with 50 or 100 μM piperine for 24 h showed a reduction of c-myc and phospho-Histone H3 (pH3) levels (Fig. 5H), consistent with a cell cycle arrest effect. We concomitantly treated SW480 cells with 10 μM XAV939, a Wnt/β-catenin signaling inhibitor, to check whether c-myc and pH3 protein levels alteration were consistent with Wnt signaling inhibition. XAV939 treatment also reduced c-myc and pH3 levels of SW480 cells.

Similarly, RKO cells were numerically increased in G0/G1 phase (Fig. 5I–M—gate M1) and decreased in G2/M phase of cell cycle in both piperine treated conditions (Fig. 5O—gate M3). Piperine treatment did not alter the number of RKO cells in S phase (Fig. 5N—gate M2). Additionally, RKO cell treatment for 24 h with 100 μM reduced c-myc levels (Fig. 5P). Curiously, western blot analysis showed a reduction of pH3 of RKO cells treated not only with 100 μM piperine but also with 10 μM XAV939, suggesting that Wnt signaling inhibition impairs RKO cell cycle progression. These data suggest that piperine treatment for 48 h impairs cell cycle progression and results in G0/G1 phase arrest.

### Piperine decreases intestinal tumor cell lines migration

**Figure 6:**
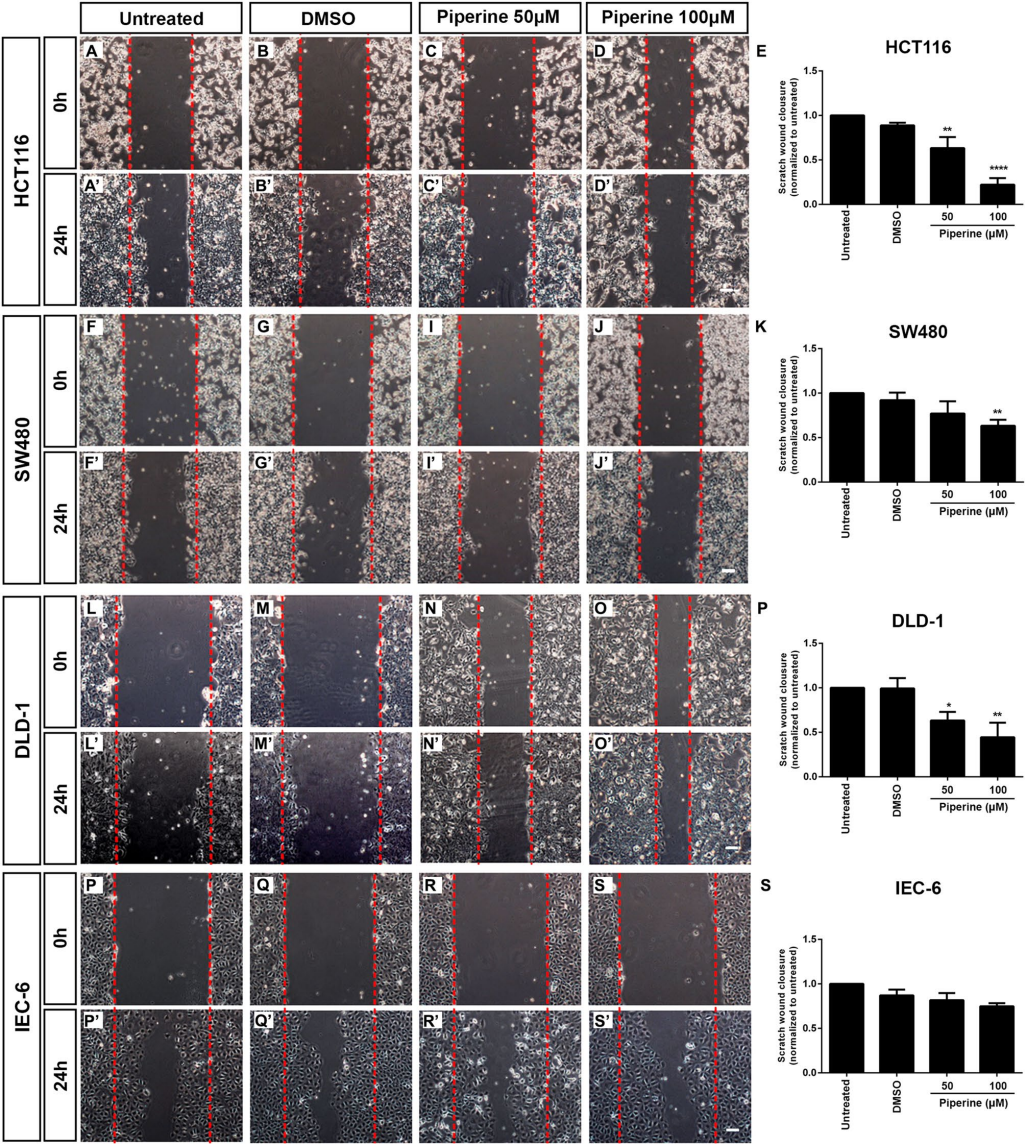
Piperine impairs colorectal tumor cell lines migration. Phase-contrast images of the scratch wound immediately after scratch of (**A**–**D**) HCT116, (**F**–**J**) SW480, (**L**–**O**) DLD-1 and (**P**–**S**) IEC-6 cell lines. Phase contrast images of scratch wound 24 h after scratch of (**A**′–**D**′) HCT116, (**F**′–**J**′) SW480, (**L**′–**O**′) DLD-1 and (**P**′–**S**′) IEC-6 cell lines. Cells were treated with DMSO, 50, 100 μM piperine, or untreated according to label. Quantification of relative scratch wound closure of (**E**) HCT116, (**K**) SW480, (**P**) DLD-1, (**S**) IEC-6 treated or not with 50, 100 µM piperine. *p < 0.05, **p < 0.01, ***p < 0.001. Scale bar = 140 μm.

One of the hallmarks of colorectal cancer is cell migration following metastasis. Once piperine displayed antitumor effects on colorectal cancer cell lines, we wondered whether it would also impair cell migration. To test this hypothesis we performed the scratch assay using HCT116, SW480, DLD-1 and IEC-6 cell lines treated with 50 or 100 μM piperine for 24 h. Piperine inhibited cell migration of every colorectal cancer cell lines tested whereas it did not affect the non-tumoral intestine cell line IEC-6 migration rate (Fig. 6P–S,P′–S′). 50 and 100 μM piperine decreased by 30% to 70% the migration rate of HCT116 cells, respectively (Fig. 6A–E,A′–D′). 50 and 100 μM piperine inhibited by 30% and 76% the migration rate of DLD-1 colorectal cancer cell line, respectively (Fig. 6L–P,L′–O′). SW480 cells were more resistant to piperine treatment, since only 100 μM piperine inhibited by 32% the cell migration rate (Fig. 6F–K,F′–J′). Intriguingly, piperine showed no effect in RKO cells after 24 h treatment. Suggesting that piperine anti-migratory effects rely on increased Wnt signaling activity.

## Discussion

The pharmacological properties of alkaloids have been extensively studied, and among them, the anti-tumor effects of these compounds were highlighted. More than ten thousand alkaloids presented pharmacological uses, including antitumor effects, however, only a fraction of this enormous number was well-studied^38^. Alkaloids are among the most important small molecules within the natural products group, and some of these were successfully developed into anticancer commercial drugs, such as Camptothecin (CPT), a topoisomerase I inhibitor^39^, besides taxol and vimblastine, which interact with tubulin^40,41^. Screenings for new molecules led to the discovery of novel alkaloids that have shown promising antineoplastic and anti-proliferative effects in different types of cancer cell lines. Among these compounds, piperine is included as one of the most studied^42^. The anti-cancer effects of piperine were shown for lung, prostate, breast, melanoma and colorectal cancers, however, very few papers showed the mechanisms of actions of piperine on cell machinery. In this study, we show that piperine inhibits Wnt/β-catenin signaling even if we activate the pathway with a constitutively activated form of β-catenin (S33A mutated) or with a constitutively activated form of TCF, independent of β-catenin binding (Fig. 1). Piperine inhibits β-catenin nuclear translocation in HCT116 colorectal cancer cell line and suppresses proliferation (Fig. 4) and migration (Fig. 6) of different colorectal cell lines with different Duke´s grade without affecting non-tumoral intestine cell line IEC-6.

It is a consensus in literature that piperine can be used as an anti-cancer compound, since it induces apoptosis in different types of cancer cell lines^35,43–45^. However, the molecular mechanisms by which piperine performs anti-cancer effects are not fully understood yet. Our proliferation and migration data suggests that piperine effect is partially due to its Wnt signaling inhibitory effect, since piperine has displayed reduced effects in cell lines that do not predominantly rely on Wnt signaling for growth (Fig. 4, 6). This hypothesis is endorsed by the absence of effect of piperine treatment on HEK293T CTNNB1 KO treated cells (Supplementary Figure S1). Nevertheless, piperine effect mechanisms probably depend on multiple pathways modulation. For example, piperine suppresses growth and migration of breast cancer cell lines with different genetic background. Breast cancer cell lines treated with piperine have increased activation of AKT pathway leading to Caspase-dependent apoptosis^44^. On the other hand, piperine treatment inhibited AKT phosphorylation and mTOR pathway in DU145 prostate cancer cell line preventing cell migration^46^.

Co-treatment of HeLa cells xenografts with piperine and Mitomycin C impaired tumor growth, decreased p-STAT3 and NF-κB and increased the apoptotic factors Bax, Bid, Caspases and PARP expression^45^. Piperine also impaired cell cycle and activated the apoptotic pathway in melanoma and prostate cancer cell lines^35,43^. Additionally, piperine inhibited STAT3 in TMK-1 gastric cancer cell line. Suppression of STAT3 and p38 MAPK in TMK-1 by piperine treatment led to inhibition of IL-6, an important cytokine that induces malignancy in gastric cancer^47^.

Several studies reported anti-invasive or anti-migratory effects of piperine in different cancer cell lines^33,44,48^. These studies showed that piperine inhibits the expression of matrix metalloproteinase (MMP) in HT1080 human fibrosarcoma cell line and 4T1 breast cancer cell line, DU145 prostate cancer and HOS and U2OS osteosarcoma cell lines. Our results show that piperine impairs migration of HCT116, SW480 and DLD-1 colorectal cancer cell lines and does not interferes with the migration rate of IEC-6 a non-tumoral intestine cell line.

Only one study evaluated the effects of piperine on Wnt signaling, however this study analyzed the effects of a co-treatment of piperine and curcumin on Wnt signaling in breast cancer cell lines^49^. Nevertheless, this study did not show on which level piperine acts. Our results show through epistasis experiments that piperine inhibits Wnt signaling activated by constitutively activate form of TCF (dnTCF4 VP16) and also inhibits β-catenin nuclear localization in HCT116, a colorectal cancer cell line that harbors a mutated β-catenin form that overactivates Wnt signaling. Piperine Wnt signaling inhibition property is probably one additional effect that contributed for tumor growth impairment reported in previous publications. Recently, two works showed that piperine binds directly to DNA. Haris and co-workers revealed the molecular mechanism of interaction of piperine with DNA showing that piperine binds to DNA minor groove^50^. Another work showed that piperine binds to various G-quadruplex DNA sequences, including the *c-myc* promoter, one of Wnt signaling pathway target genes^51^. These recent findings, together with our epistasis experiment using dnTCF4 VP16 indicate that piperine could act through different pathways and could even have different targets in the Wnt/β-catenin signaling cascade. Our data suggests that piperine inhibits the translocation of β-catenin to the nucleus and might suppress the binding of TCF/LEF to the DNA, or even by direct binding to the promoter and downregulating Wnt target genes.

Nowadays, natural compounds with multiple targets are being revisited and studied for anti-cancer effects. Piperine displays anti-cancer effects in different cancer cell lines, probably by interfering with Wnt/β-catenin signaling, among others. Thus, a new path is opened for understanding the molecular mechanisms by which alkaloids suppress tumor progression.

## Materials and methods

### Cell lines, chemicals and reagents

All cell lines were maintained in DMEM F12 culture medium supplemented with 10% fetal bovine serum and 100U/mL and 100 µg/mL penicillin/streptomycin (Gibco, Life Technologies Carlsbad, CA, USA) at 37 °C and 5% CO_2_. Dimethylsulfoxide (DMSO), anti-β-catenin and anti-α-tubulin were purchased from Sigma-Aldrich (St. Louis, MO, USA). Secondary antibodies were purchased from Life Technologies (CA, USA). Cell lines used were HEK293T, L-cell, L-Wnt3a, HCT116, DLD-1, SW480 and IEC-6 (ATCC). Wnt signalling reporters cell lines, RKO pBAR/*Renilla* and SW480-pBAR/*Renilla* were a kindly donated by Dr. Xi He (Boston Children´s Hospital). The alkaloid piperine used in this study was extracted from *Piper nigrum*, purified and identified at Research Institute for Natural Products in the Federal University of Rio de Janeiro (IPPN-UFRJ-Brazil).

### Isolation of amides

Piperine was isolated from the seeds of *Piper nigrum*^52^. Methanolic extracts from seeds of *P. nigrum* were submitted to successive column chromatography using silica gel and gradient of solvents with increasing polarity. Piperine was isolated as yellow cristal and its identity was confirmed by comparison of the ^1^H NMR data with the literature^20^.

### High performance liquid cromatography

The experiment was carried out in a high efficiency liquid chromatograph (HPLC) of the Shimadzu-LC20AD brand with photodiode array detection (CLAEPDA), Kromasil 100-5C18 column (4.6 mm by 250 mm), SPD-M20A detector (Diode Array) at 345 nm wavelength and 1 mL/minute constant flow. Piperine sample was diluted in acetonitrile. Automatic injection programming was performed so that the injected sample volume corresponded to 20 μL. Mobile phases used were: 60% acetonitrile and 40% water in the isocratic method. The running time was 15 min. The retention time band 7.15 min (Supplementary Figure S2) corresponded to the major compound, displaying an area of 98.79% (Supplementary Table S1). We can infer that piperine purity is 98.79%.

### High resolution electrospray ionization mass spectrometry analysis

HRESIMS was carried out in a Bruker Solarix XR 7Tesla mass spectrometer available at the MASS SPECTROMETRY CENTER OF BIOMOLECULES (CEMBIO), UFRJ. To obtain the mass spectra the sample was dissolved in high purity methanol. The data was acquired in the positive mode using the Electrospray ionization method (ESI) on a scale of 96.77 to 1,200 m/z. The spectrum was analyzed using the mMass 5.5.0 software. The calculated [M + H] + molecular mass corresponding to piperine is 286.1438 and the found mass is 286.1432 (Supplementary Figure S3). This indicates that the compound used in this work in, in fact, piperine.

### Wnt-luciferase reporter assays

1.2 × 10^4^ cells were cultured on 96-well plates until confluence. RKO-pBAR/*Renilla* cells were treated with piperine 10, 30, 50 and 100 μM in the presence of Wnt3a conditioned medium (Wnt3a CM). L-cell conditioned medium (L-cell CM) was used as negative control. DMSO 0.2% was also added as the vehicle control. In SW480-pBAR/*Renilla* cells there was no need for Wnt3a treatment since the pathway is overactivated in this lineage. After 24 h of treatment, Firefly and *Renilla* luciferase activities were detected according to the manufacturer’s protocol (Dual Luciferase Reporter Assay System, Promega). 1.2 × 10^4^ HEK293T cells were cultured on 96-well plates until they reach 70% confluence, cells were transfected with lipofectamine 3,000 (Lipofectamine™ 3,000 Reagent; Invitrogen) and 100 ng TOP-Flash plasmid, 10 ng CMV-Renilla-luciferase, and 100 ng of the vector pCS2, or pCS2-β-catenin WT, or pCS2-β-catenin S33A, or pCS2-dnTCF4 VP16. dnTCF4-VP16 construct expression results in the translation of a fusion protein that contains the transcription activation domain of herpes simplex virus (VP16) and a truncated TCF4 that cannot bind to β-catenin (dnTCF4)^53,54^. dnTCF4 itself is a dominant negative form of TCF4, however, when fused with VP16, it becomes constitutively active, since it activates the Wnt/β-catenin in a β-catenin independent manner. 18 h after transfection, cells were treated for 24 h, using 10, 30, 50 or 100 μM of piperine. *Firefly* and *Renilla* luciferase activities were detected according to the manufacturer’s protocol (Dual Luciferase Reporter Assay System, Promega).

### MTT assay

3-(4,5-Dimethylthiazol-2-yl)-2,5-diphenyl tetrazolium bromide (MTT) (Sigma-Aldrich) was used to assay mitochondrial activity in viable cells. Cells were plated at a concentration of 1.2 × 10^4^ cells/well in 96-well tissue culture plates and cultured for 24 h before treatment with 20, 30, 50, 100 or 200 μM piperine or the vehicle DMSO for 0, 24, 48, or 72 h. MTT was added to each well at a final concentration of 0.25 mg/mL for 1 h before cell harvesting. The formazan reaction product was dissolved with DMSO and quantified spectrophotometrically at 570 nm using a UV–visible system. (Modulus II microplate multimode reader).

### Cell proliferation assay

For cell proliferation assay, 5.0 × 10^4^ cells were plated on the previous day and treated with 50, 100 or 200 μM of piperine for 24 h. Click-iT EdU (Life Sciences) assay was performed according to manufacturer’s protocol. DMSO was used as a vehicle to solubilize the alkaloid and was added to control cultures conditions at 1%.

### Cell cycle analysis

RKO and SW480 cell lineages were cultured for 48 h with medium alone (untreated), DMSO, 50 µM piperine or 100 µM piperine. Subsequently, supernatant of each condition was collected, and cells were detached with trypsin at room temperature. After centrifugation, cells were resuspended in 0.5 mL ice-cold Vindelov solution^55^, containing 0.1% Triton X-100, 0.1% citrate buffer and 0.1 mg/mL RNase and 50 μg/mL propidium iodide (Sigma-Aldrich, USA). After 15 min incubation, we analyzed the cell suspension for DNA content by flow cytometry using a FACSCalibur flow cytometer (Becton Dickinson, USA). The relative proportions of cells with DNA content diploid G0/G1 (2n), S phase (> 2n but < 4n), and G2/M phase (4n) were acquired and analyzed using CellQuest software. Cell dissociation procedure does not affect fluorescence under the experimental conditions that were used in this study or in any others of which we are aware.

### Immunostaining

RKO pBAR-*Renilla* cells were fixed in 4% paraformaldehyde, washed with PBS, and permeabilized with 0.1% Triton X-100. Samples were then blocked for 1 h with 5% normal goat serum (Gibco). A rabbit anti-β-catenin (1:200) primary antibody was incubated overnight. Specific secondary antibody Alexa 546 (Alexa Fluor ®546 Goat Anti-Rabbit IgG; Invitrogen) (1:2000) were incubated for 1 h at room temperature. After PBS washes, DAPI staining (4,6-diamidino-2-phenylindole) (Sigma) was performed for 10 min, and then slides were mounted with Fluormount (Sigma) and observed at confocal microscope (Leica TCS SP5).

### Western blotting

Lysate samples from piperine treated cells at 50 and 100 μM were harvested in Triton buffer (150 mM NaCl, 50 mM Tris, 1% Triton X-100, 1 mM EDTA, 10% Glycerol, pH 7.5). Protein was quantified by the Bradford method, and 20 μg of the total lysate was loaded in 10% sodium dodecyl sulfate–polyacrylamide gel electrophoresis and transferred to a polyvinylidene fluoride membrane (Immobilon-E, Millipore). Membranes were pre-blocked in 5% nonfat dry milk in Tris-buffered saline, 0.001% Tween 20 for 1 h and then incubated overnight with anti-β-catenin (1:1,000, Sigma, C2206), anti-α-tubulin (1:1,000, Sigma, T5168), anti-c-Myc (1:1,000, CST, #5,605), anti-phospho Histone-H3 (1:1,000, CST, #3,377), anti-β-actin (1:2000, SCBT, sc-47778) primary antibodies previously diluted in Tris-buffered saline, 0.001% Tween 20, 1% nonfat milk. Secondary antibodies conjugated with horseradish peroxidase (1:3,000) diluted in the same buffer were used to probe the membranes, and the reaction was visualized using SuperSignal West Pico and SuperSignal West Femto chemiluminescent substrate.

### CRISPR-Cas9 CTNNB1 knockout

The sgRNA designed to target “GAGTGGTAAAGGCAATCCTG” was cloned into the lentiCRISPRv2 according to Feng Zhang laboratory manual. HEK293T cells were transfected with the lentiCRISPRv2 cloned plasmid and clonally expanded with selection medium containing puromycin. CTNNB1 knockout (KO) efficiency was examined by Western blot. LentiCRISPRv2 plasmid was obtained from Addgene (Plasmid #52961).

### Scratch assay

HCT116, SW480, DLD-1 and IEC-6 cell cultures were cultivated in 12-well plates until confluence and treated with 50 or 100 μM of piperine, together with Ara-C, an inhibitor of cellular proliferation. A line down the center of each well was scraped with a pipette tip. Images were taken at 0 h and 24 h after treatment, scratches area were measured, and wound closure was calculated by subtraction of the area measured after 24 h of incubation by the initial scraped area. Each experiment was carried out in triplicate, and three fields were counted per well.

### Statistical analysis

Each experiment was performed at least three times. The luciferase and MTT assays were performed in triplicate. Cell staining quantification was performed by counting the number of DAPI and the number of non-nuclear and nuclear β-catenin stained cells in randomly chosen microscope fields, and then the percentage of nonnuclear and nuclear cells of the total cells was calculated. Statistical analysis were performed using the unpaired Student’s t test. In the MTT assays, we used two-way ANOVA following a Bonferroni post-test (GraphPad Prism version 6.00, GraphPad Software Inc., La Jolla, CA, USA). Standard error and significance (*p* value) were determined by paired Mann–Whitney test (GraphPad Prism Software, version 6.00).

## Supplementary information

Supplementary file1 (PDF 882 kb)

## Data Availability

The datasets generated during and/or analyzed during the current study are available from the corresponding author on reasonable request.
