# Supplementary material for: Piperine suppresses the Wnt/β-catenin pathway and has anti-cancer effects on colorectal cancer cells
Source: Sci Rep. 2020 Jul 15;10:11681. doi: 10.1038/s41598-020-68574-2 (PMC7363889; doi:10.1038/s41598-020-68574-2)
Supplement: Supplementary file 1 — Supplementary file1 (PDF 882 kb) [file 41598_2020_68574_MOESM1_ESM.pdf]

Supplementary Info File

# Title: Piperine suppresses the Wnt/β-catenin pathway and has anti-cancer effects on colorectal cancer cells

Authors: Gracielle C. de Almeida[1], Luiz F. S. Oliveira[1], Danilo Predes[1], Harold H. Fokoue[2,3], Ricardo M. Kuster[4], Felipe L. Oliveira[1], Fabio A. Mendes[1] *, Jose G. Abreu[1] *.

Affiliations: [1] Instituto de Ciências Biomédicas, Programa de Biologia Celular e do Desenvolvimento, Universidade Federal do Rio de Janeiro, Rio de Janeiro, RJ, Brazil; [2] Instituto Nacional de Ciência e Tecnologia de Fármacos e Medicamentos (INCT-INOFAR; http://www.inct-inofar.ccs.ufrj.br/), Laboratório de Avaliação e Síntese de Substâncias Bioativas (LASSBio®, http://www.lassbio.icb.ufrj.br) Universidade Federal do Rio de Janeiro, CCS, Cidade Universitária, Rio de Janeiro, RJ, Brazil. [3] Programa de Pós-Graduação em Farmacologia e Química Medicinal, Instituto de Ciências Biomédicas, Universidade Federal do Rio de Janeiro, Avenida Carlos Chagas Filho, 373, Ilha do Fundão, 21941-912, Rio de Janeiro, RJ, Brazil. [4] Instituto de Pesquisas em Produtos Naturais, Universidade Federal do Rio de Janeiro, Rio de Janeiro, Brazil.

Correspondence to: Fabio A. Mendes, mendes@icb.ufrj.br and Jose G. Abreu, garciajr@icb.ufrj.br

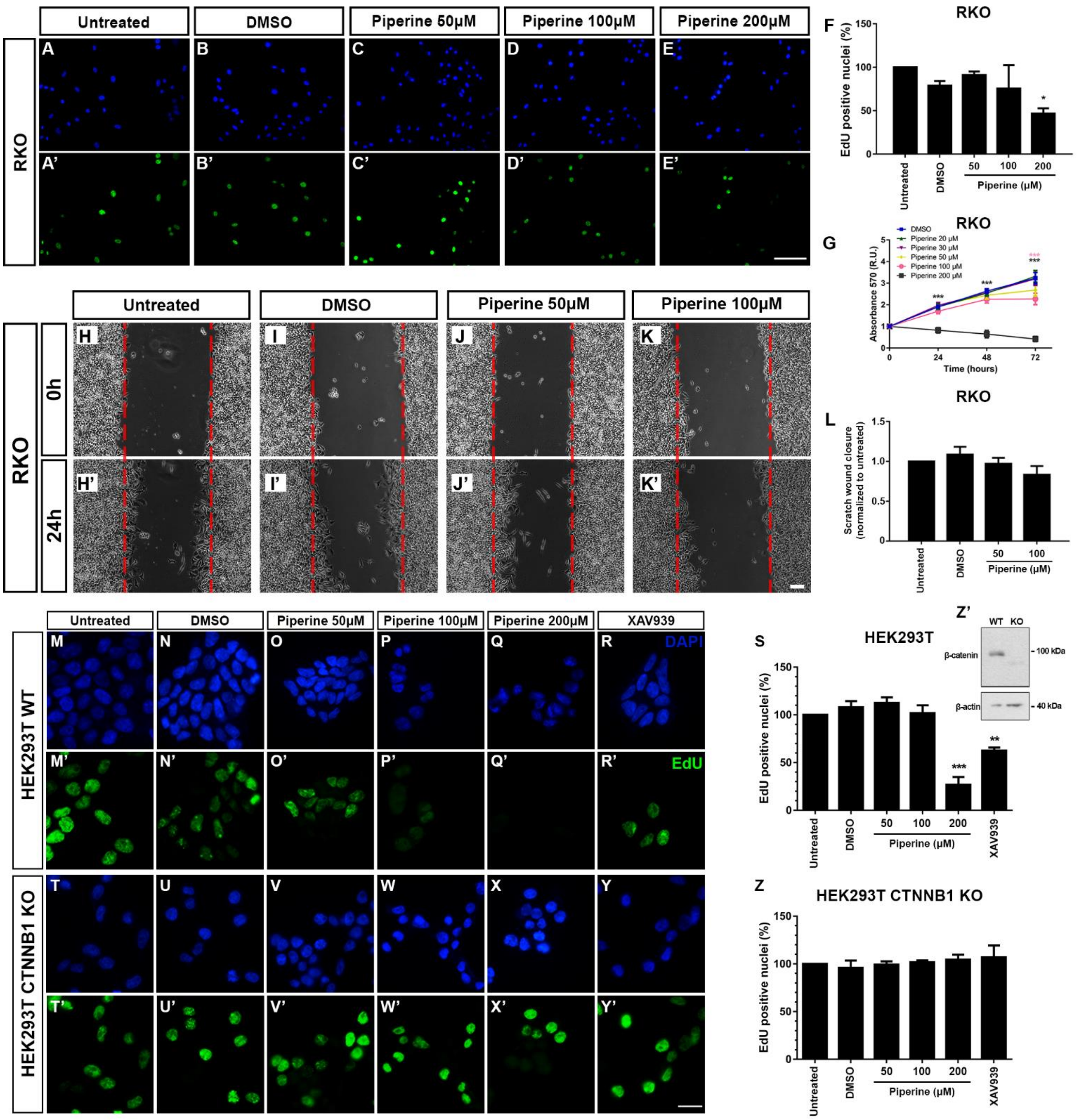


**Supplementary Figure S1. Piperine has less effect on a colorectal cell line without overactive Wnt signaling.**

(A-F) Click-iT EdU assay of RKO cells untreated or treated with DMSO, 50, 100 or 200 µM piperine. Scale bar = 50 µm (F) Graph shows that 200 µM piperine has effect on RKO cell lines proliferation. (G) MTT assay shows that RKO cells has reduced viability after piperine treatment. (H-L) Scratch assay of RKO cells untreated or treated with DMSO, 50, 100 µM piperine. Scale bar = 140 µm. (L) Graph shows that piperine has no effect on RKO cells migration after 24h treatment. (M-S) Click-iT EdU assay of HEK293T cell line untreated or treated with DMSO, 50,

100 or 200 µM piperine. (T-Z) Click-iT EdU assay of HEK293T CTNNB1 KO cell line untreated or treated with DMSO, 50, 100 or 200 µM piperine. Scale bar = 20 µm (Z') Western blot assay showing absence of β-catenin band after CRISPR mediated knockout. Graphs shows mean and SEM. *$p<0.05$, **$p<0.01$, ***$p<0.001$.

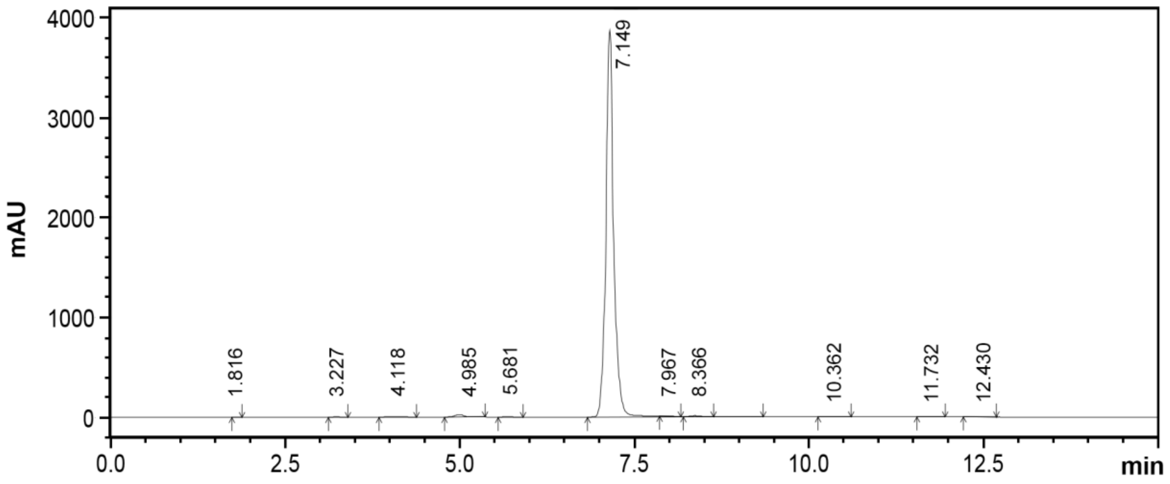


**Supplementary Figure S2: Piperine HPLC analysis**.
Data: UVvis ($\lambda_{max}$) = 345 nm , Retention time: 7.149 min; purity: 98.79%. (Mobile phase: Acetonitrile: 60% / Water: 40%).

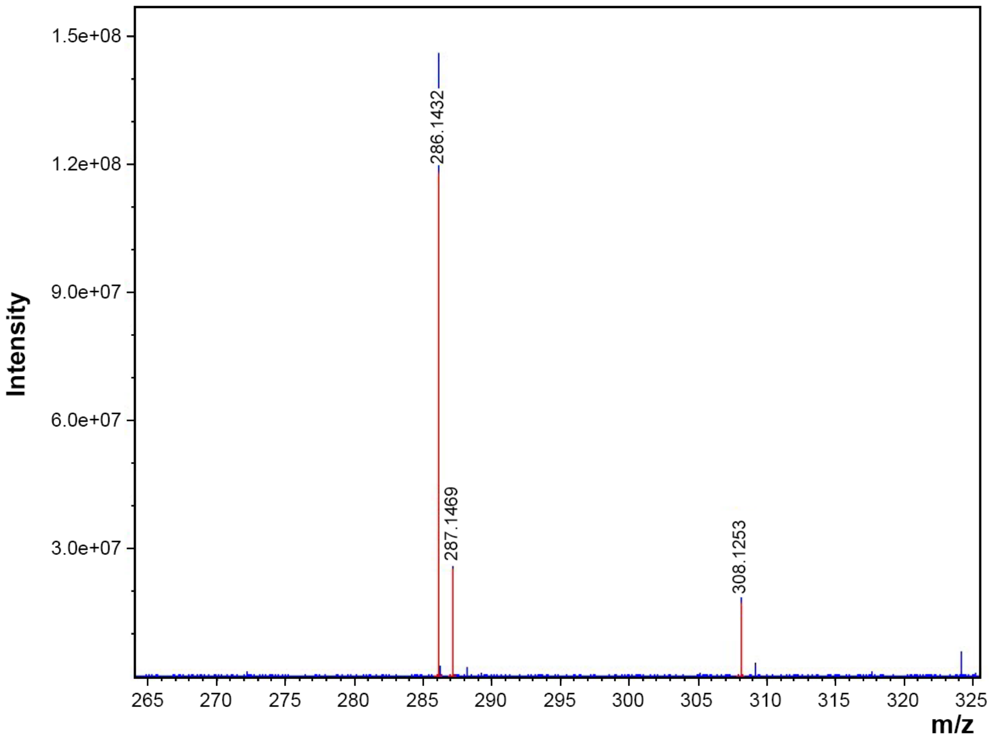


**Supplementary Figure S3: Piperine high resolution electrospray ionization mass spectrometry analysis.**

HRESIMS: m/z calculated for C17H21NO3+[M+H]+ = 286.1438; found = 286.1432. m/z calculated for C17H21NNaO3+[M+Na]+ = 308.1257; found = 308.1253. m/z: Mass to charge ratio.

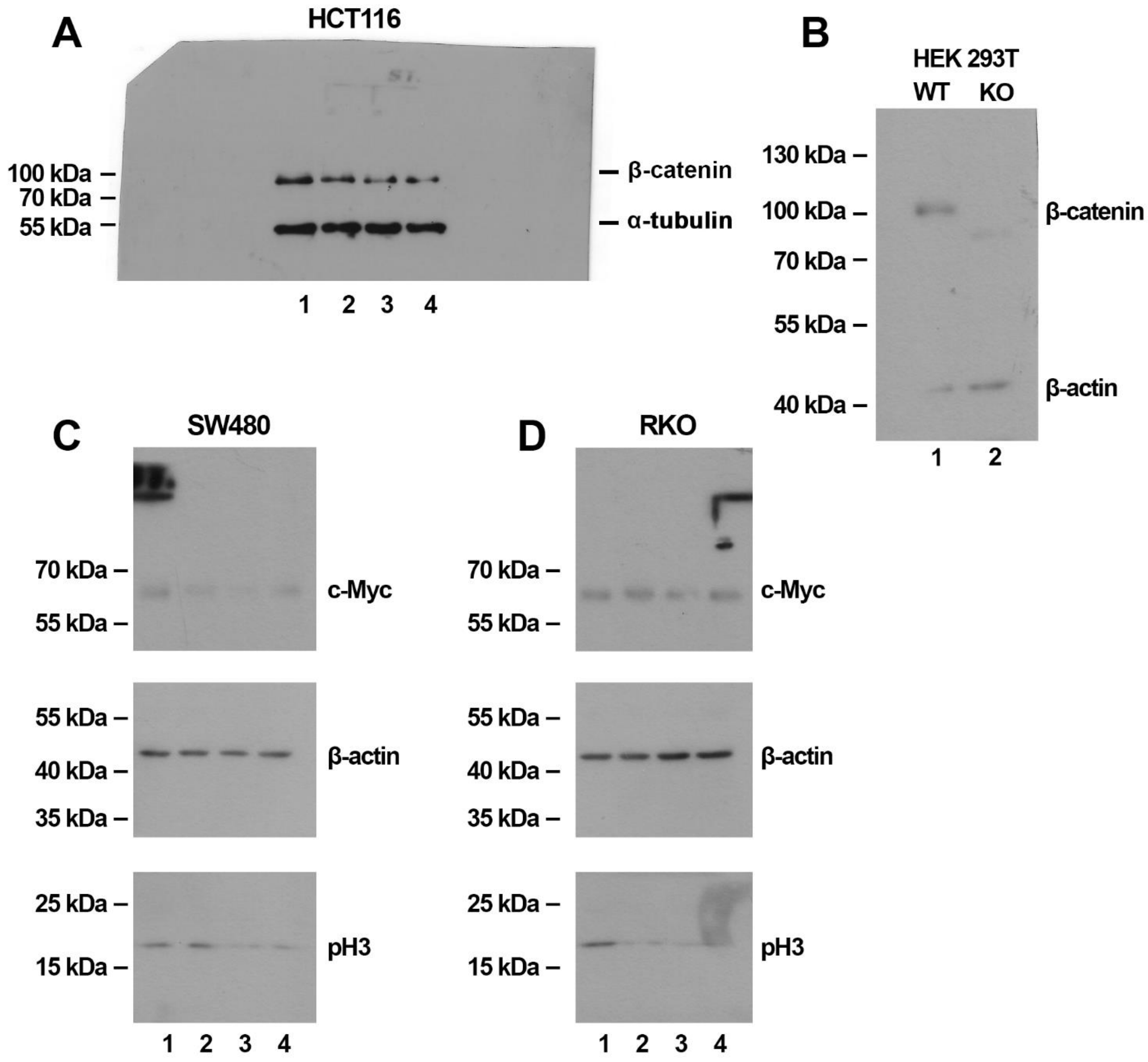


**Supplementary Figure S4: Raw immunoblot data.**

(A) Raw immunoblot data from HCT116 cell lysate shown in Figure 2. 1: Untreated; 2: DMSO; 3: 50μM piperine; 100μM piperine. (B) Raw immunoblot data from HEK293T cell lysate shown in Figure S1. 1: HEK293T WT, 2. HEK293T CTNNB1 KO. Raw immunoblot data from (C) SW480 or (D) RKO cell lysate shown in Figure 5: 1: DMSO; 2: 50μM piperine; 3: 100μM piperine; 4: 10μM XAV939. Anti-β-catenin (1:1000, Sigma, C2206), anti-α-tubulin (1:1000, Sigma, T5168), anti-c-Myc (1:1000, CST, #5605), anti-phospho Histone-H3 (1:1000, CST, #3377), anti-β-actin (1:2000, SCBT, sc-47778).

| Peak n# | Retention Time (min) | Area | Height | Area (%) | Height (%) |
|---|---|---|---|---|---|
| 1 | 1.816 | 1128 | 184 | 0.004 | 0.005 |
| 2 | 3.227 | 1607 | 198 | 0.005 | 0.005 |
| 3 | 4.118 | 19044 | 2089 | 0.062 | 0.053 |
| 4 | 4.985 | 204042 | 23941 | 0.666 | 0.613 |
| 5 | 5.681 | 1686 | 204 | 0.005 | 0.005 |
| 6 | 7.149 | 30283620 | 3868230 | 98.788 | 98.964 |
| 7 | 7.967 | 26178 | 2823 | 0.085 | 0.072 |
| 8 | 8.366 | 106734 | 10196 | 0.348 | 0.261 |
| 9 | 10.362 | 6370 | 486 | 0.021 | 0.012 |
| 10 | 11.732 | 1769 | 158 | 0.006 | 0.004 |
| 11 | 12.430 | 2877 | 205 | 0.009 | 0.005 |
| Total | | 30655056 | 3908714 | 100.000 | 100.000 |

**Supplementary Table S1: Peak table of piperine HPLC analysis.**
